# Identification and Preclinical Pharmacology of the **γ**-Secretase Modulator BMS-869780

**DOI:** 10.1155/2014/431858

**Published:** 2014-07-08

**Authors:** Jeremy H. Toyn, Lorin A. Thompson, Kimberley A. Lentz, Jere E. Meredith, Catherine R. Burton, Sethu Sankaranararyanan, Valerie Guss, Tracey Hall, Lawrence G. Iben, Carol M. Krause, Rudy Krause, Xu-Alan Lin, Maria Pierdomenico, Craig Polson, Alan S. Robertson, R. Rex Denton, James E. Grace, John Morrison, Joseph Raybon, Xiaoliang Zhuo, Kimberly Snow, Ramesh Padmanabha, Michele Agler, Kim Esposito, David Harden, Margaret Prack, Sam Varma, Victoria Wong, Yingjie Zhu, Tatyana Zvyaga, Samuel Gerritz, Lawrence R. Marcin, Mendi A. Higgins, Jianliang Shi, Cong Wei, Joseph L. Cantone, Dieter M. Drexler, John E. Macor, Richard E. Olson, Michael K. Ahlijanian, Charles F. Albright

**Affiliations:** ^1^Exploratory Biology and Genomics, Bristol-Myers Squibb Research and Development, 5 Research Parkway, Wallingford, CT 06492, USA; ^2^Discovery Chemistry, Bristol-Myers Squibb Research and Development, 5 Research Parkway, Wallingford, CT 06492, USA; ^3^Pharmaceutical Candidate Optimization, Bristol-Myers Squibb Research and Development, 5 Research Parkway, Wallingford, CT 06492, USA; ^4^Preclinical Sciences, Alexion Pharmaceuticals, Inc 352 Knotter Drive, Cheshire, CT 06410, USA; ^5^Lead Discovery and Lead Profiling, Bristol-Myers Squibb Research and Development, 5 Research Parkway, Wallingford, CT 06492, USA; ^6^High Throughput Biology, Boehringer Ingelheim, 900 Ridgebury Road, Ridgefield, CT 06877, USA; ^7^Stratford High School, 45 North Parade, Stratford, CT 06615, USA; ^8^External Research Solutions, WWMC, Pfizer World Wide Research & Development, Eastern Point Road, Groton, CT 06340, USA; ^9^Arvinas Inc, 5 Science Park, New Haven, CT 06511, USA; ^10^Discovery Analytical Sciences, Bristol-Myers Squibb Research and Development, 5 Research Parkway, Wallingford, CT 06492, USA; ^11^Department of Pharmacokinetics, Dynamics and Metabolism, Pfizer World Wide Research & Development, Eastern Point Road, Groton, CT 06340, USA

## Abstract

Alzheimer's disease is the most prevalent cause of dementia and is associated with accumulation of amyloid-*β* peptide (A*β*), particularly the 42-amino acid A*β*1-42, in the brain. A*β*1-42 levels can be decreased by *γ*-secretase modulators (GSM), which are small molecules that modulate *γ*-secretase, an enzyme essential for A*β* production. BMS-869780 is a potent GSM that decreased A*β*1-42 and A*β*1-40 and increased A*β*1-37 and A*β*1-38, without inhibiting overall levels of A*β* peptides or other APP processing intermediates. BMS-869780 also did not inhibit Notch processing by *γ*-secretase and lowered brain A*β*1-42 without evidence of Notch-related side effects in rats. Human pharmacokinetic (PK) parameters were predicted through allometric scaling of PK in rat, dog, and monkey and were combined with the rat pharmacodynamic (PD) parameters to predict the relationship between BMS-869780 dose, exposure and A*β*1-42 levels in human. Off-target and safety margins were then based on comparisons to the predicted exposure required for robust A*β*1-42 lowering. Because of insufficient safety predictions and the relatively high predicted human daily dose of 700 mg, further evaluation of BMS-869780 as a potential clinical candidate was discontinued. Nevertheless, BMS-869780 demonstrates the potential of the GSM approach for robust lowering of brain A*β*1-42 without Notch-related side effects.

## 1. Introduction

Alzheimer's disease (AD) is the most prevalent cause of dementia. More than 35 million people have dementia worldwide and the prevalence is expected to double in the next 20 years [1]. Medicines are available for treatment of symptoms but provide limited benefit and do not prevent AD [2]. The cause of AD is not completely understood, but a widely held view of its pathogenesis is based on the amyloid hypothesis. Accumulation and aggregation of the toxic amyloid-*β* peptide (A*β*), particularly the 42-amino acid form A*β*1-42 [3], initiates neuronal dysfunction that eventually leads to brain atrophy, dementia, and death [4, 5]. A*β* is naturally produced in the brain by proteolytic processing of a type I transmembrane protein, the amyloid-*β* precursor protein (APP). APP is processed by the *β*-site APP cleaving enzyme (BACE), releasing a secreted ectodomain and a membrane-anchored C-terminal fragment (APP-CTF*β*). Subsequent cleavage of APP-CTF*β* within the transmembrane domain by *γ*-secretase then produces a cytosolic intracellular domain (AICD) and A*β*, which is secreted. In addition, a fraction of APP is cleaved by *α*-secretase at a site within the A*β* sequence to produce APP-CTF*α*, which is subsequently cleaved by *γ*-secretase to produce nonamyloidogenic peptides [6]. Compounds targeting either BACE or *γ*-secretase have been tested in clinical trials, but adequate A*β*-lowering at tolerated doses in patients has been a challenge [7].

*γ*-Secretase is a lipid bilayer-embedded aspartyl protease consisting of four core subunits; nicastrin, Aph-1, Pen-2, and presenilin. Presenilin carries the active site aspartyl residues, whereas the other subunits play ancillary roles in enzymatic activity and maturation [6, 8, 9]. Structural studies of *γ*-secretase using electron micrographic image analysis and biochemical methods to map locations of amino acid residues suggest a compact structure with the active site contained within a hydrophilic chamber surrounded by transmembrane domains [10–14]. High resolution structure of *γ*-secretase has not been reported, but, based on analogy to X-ray crystallography of a presenilin homolog [15], it seems likely that the active site aspartates would be located within an intramembrane pore surrounded by the transmembrane domains of presenilin. The biological role of *γ*-secretase involves the proteolytic cleavage of transmembrane domains of at least 80 different protein substrates, including APP and the Notch family [16]. Although the physiological significance of substrate processing is unknown in most cases, *γ*-secretase cleavage of Notch and production of the Notch intracellular domain (NICD) are critical for adult cell differentiation in the immune system and gastrointestinal tract [17, 18]. Inhibitors of *γ*-secretase (GSI) can be effective for A*β* lowering; however Notch inhibition likely contributes to dose-limitation [19].

A class of small molecules that avoid Notch inhibition are the *γ*-secretase modulators (GSM), which, like GSIs, target presenilin [20–24]. In contrast to GSIs, which inhibit A*β* production, GSMs have relatively little overall effect on A*β* production. Instead, GSMs change the lengths of A*β* peptides produced, causing decreased amounts of longer peptides, such as A*β*1-40 and A*β*1-42, and increased amounts of shorter peptides, such as A*β*1-37 and A*β*1-38 [25]. GSMs have analogous effects on other *γ*-secretase substrates, including Notch, causing a shift from the longer N*β*1-25 to the shorter N*β*1-21 Notch-derived peptides [26, 27]. The shift from longer to shorter A*β* peptides was first described for several nonsteroidal anti-inflammatory drugs (NSAID), which have low potency GSM activity [28], and was subsequently found in other small molecules and natural products [25]. The effect of GSMs is thought to result from allosteric stimulation of the stepwise cleavage mechanism of *γ*-secretase. The stepwise mechanism initiates with an endopeptidic cleavage of APP-CTF near the cytosolic face of the lipid bilayer, at either position 48 or position 49 (using the conventional amino acid numbering starting at position 1 for the aspartyl residue at the N-terminus of A*β*). Subsequently, *γ*-secretase carries out a series of carboxypeptidase-like cleavages at three or four amino acid intervals thereby producing tripeptides or tetrapeptides and A*β* peptides of different lengths [29, 30]. Typically, the 40-amino acid long A*β*1-40 is the major product, but lesser amounts of other A*β* peptides such as A*β*1-38 and A*β*1-42 are also produced. For example, A*β*1-40 biogenesis appears to require four cycles of APP-CTF cleavage at positions 49, 46, 43, and 40, resulting in the release of three tripeptides and one AICD (amino acids 50–99) for each A*β*1-40 peptide produced. Likewise, production of the A*β*1-42 peptide is associated with a series of cleavages that start at position 48 of APP-CTF [30, 31]. In the presence of a GSM, *γ*-secretase carries out an increased number of carboxypeptidase cycles per molecule of APP-CTF substrate, resulting in shorter A*β* peptides without substantially affecting the overall amount of A*β* produced [27, 32].

Despite having low potency, NSAID GSMs such as flurbiprofen were reported to lower brain A*β*1-42 in rodents [33, 34]. However, in clinical trials, flurbiprofen (tarenflurbil) was found to have no effect on A*β*1-42 in cerebrospinal fluid even at high doses [35]. High potency GSMs have also entered early stage clinical trials, but effects on A*β*1-42 in cerebrospinal fluid have not been reported [36, 37]. Many further GSMs have been evaluated* in vitro* and in animal studies, which, like the NSAID GSMs, do not inhibit Notch or other *γ*-secretase substrates [25, 38–51]. In addition, chronic dosing of GSMs has been reported to ameliorate plaque pathology [39, 45, 52] and enhance cognition in APP transgenic mice [48, 52, 53]. Thus, GSMs are capable of lowering brain A*β*1-42 and are expected to avoid the dose limitations of GSIs that are due to Notch-related side effects. A number of studies have reported at least some of the systemic exposure data associated with brain A*β*1-42 lowering, thus giving an idea of active exposures and doses for GSM [39–42, 44–46, 52]. However, preclinical predictions relating to safety margins or human dose have not been reported. Furthermore, the active exposures and doses in rodents are high, making it a matter of conjecture whether or not any of the currently published GSMs are likely to exhibit adequate safety margins or clinically acceptable doses. Here we describe the pharmacological properties of the GSM BMS-869780, a potent bicyclic triazole GSM [54, 55], and the quantitative predictions of human dose and off-target side effects. While these outcomes prevented the development of BMS-869780, they put in perspective the extent of further enhancements in drug-like properties that would be necessary to justify clinical testing of a future GSM.

## 2. Materials and Methods

### 2.1. Compounds

**Figure 1:**
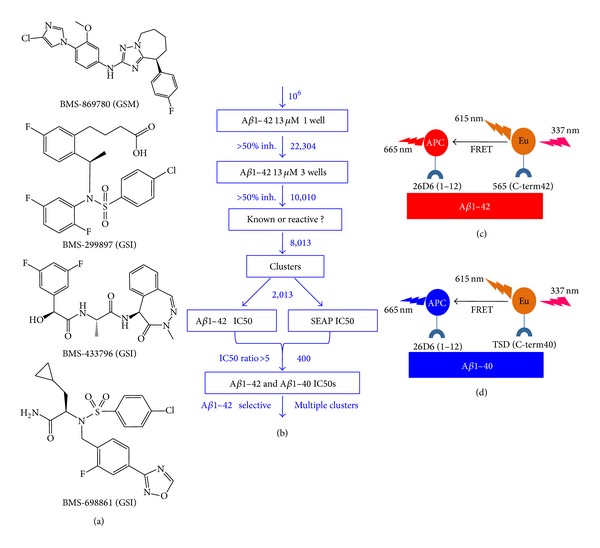
(a) Chemical structures of the compounds used in this study are shown. (b) Overview of the HTS and subsequent triage of compounds summarizes experimentation steps in boxes, with outcomes indicated beside the arrows. Costs of reagents and disposables were a major consideration in the design, particularly the initial screen of 10^6^ samples. (c) Principle of the A*β*1-42 immunoassay; simultaneous binding of monoclonal antibody conjugates 252-APC and 565-Eu (specific for C-terminus of A*β*1-42) to A*β*1-42 leads to FRET-based emission at 665 nm. The ratio of emission at 665 nm to fluorescence at 615 nm represents the level of A*β*1-42 in the sample. (d) Principle of the A*β*1-40 immunoassay; same as described above for the A*β*1-42 immunoassay, except that the monoclonal antibody conjugate TSD-Eu (specific for C-terminus of A*β*1-40) was used in place of 565-Eu.

The GSM, BMS-869780 [54, 55], and the GSIs BMS-299897 [56] and BMS-433796 [57] have been reported previously. The GSI BMS-698861 is described in a BMS patent [58]. Chemical structures are shown in Figure 1(a).

### 2.2. Cell Cultures

H4-APPsw cell cultures were maintained on Dulbecco's modified Eagle's medium (DMEM) supplemented with L-glutamine (2 mM), fetal bovine serum (10%), and G418 (100 *μ*g/mL). For IC_50_ determinations, cells were harvested, resuspended in DMEM supplemented with 0.0125% bovine serum albumin, and dispensed into 384-well plates (1.5 × 10^4^ cells per well). A*β*1-42 and A*β*1-40 assays, and Notch inhibition assays, were carried out as described previously [59]. Mouse embryonic fibroblasts deficient in PS1 and PS2 (MEF dKO) [60] were passaged twice per week in DMEM/F-12 medium, consisting of a 1 : 1 mixture of DMEM and F-12 nutrient mixture supplemented with 10% fetal bovine serum, penicillin, and streptomycin. For expression of human presenilin-1 in MEF dKO cells, the full length human presenilin-1 cDNA open reading frame was cloned between the BamHI and XhoI sites of the vector pcDNA5/FRT (Invitrogen), placing presenilin-1 expression under the control of the CMV promoter. The M146V mutated allele was introduced into the presenilin-1 expression construct by polymerase chain reaction using the oligonucleotide primers 5′-CAGTGTCATTGTTGTCGTGACTATCCTCCTGGTGG-3′ and 5′-CCACCAGGAGGATAGTCACGACAACAATGACACTG-3 (QuikChange kit, Invitrogen). MEF dKO cultures were cotransfected with DNA constructs expressing APP-CTF, encoding the C-terminal 99 amino acids of APP [61] and either the human presenilin-1 or presenilin-1 M146V allele. Transfected cultures were incubated overnight at 37°C in 5% CO_2_ atmosphere and then harvested, resuspended in DMEM/F-12 medium, dispensed into 96-well culture plates, and incubated for 6 hours at 37°C in 5% CO_2_ atmosphere. Culture medium was then replaced with Ultraculture serum free medium (Lonza, Rockland, ME) with or without compounds at a range of concentrations and incubated overnight.

### 2.3. A*β* Antibodies and Conjugates

Anti-A*β* monoclonal antibodies and their epitopes used in this study were 4G8 (A*β*17-24; Covance), 252Q6 (rodent A*β*1-12; Invitrogen), D2A6H (A*β*37 C-terminal; Cell Signaling, catalog number 12356BF), TSD (A*β*40 C-terminal), 26D6 (human A*β*1-12), and 565 (A*β*42 C-terminal; Bristol-Myers Squibb). The covalent antibody-fluorophore conjugates were made at Perkin-Elmer, including TSD-Europium cryptate (TSD-Eu), 565-Europium cryptate (565-Eu), and 26D6-allophycocyanin (26D6-APC). Streptavidin-horseradish peroxidase (SA-HRP) and 4G8-biotin conjugates were from Covance. Horseradish peroxidase (HRP) conjugates of 565, 26D6 and 252Q6 (565-HRP, 26D6-HRP and 252Q6-HRP) were made using preactivated HRP (Easylink, Zymed/Invitrogen). The 6E10-sulfo-tag conjugate was from Mesoscale Discovery (catalog number K15148E-1), and the 252Q6-sulfo-tag conjugate was made using a kit (Mesoscale Discovery catalog number R91AN-1).

### 2.4. Immunoassays

IC_50_ determinations for A*β*1-42 and A*β*1-40 in H4-APPsw cultures in 384-well format were determined using homogeneous time-resolved fluorescence immunoassays as previously described [59]. The principle of these assays is illustrated in Figures 1(c) and 1(d). In other experiments using H4-APPsw cultures, A*β* was quantified by ELISA; for A*β*1-42 the combination of monoclonal antibodies was 26D6 and 565-HRP; for A*β*1-40 it was TSD and 26D6-HRP, and for A*β*1-*x* it was 4G8 and 26D6-HRP. In some experiments a novel 4-plex A*β* electrochemiluminescence immunoassay was used (Mesoscale Discovery catalog number N45ZA-1). Briefly, the 4-plex was carried out in 96-well format, with 4 separate spots of capture antibodies in each well. The 96-well plates were prepared by the manufacturer, with spots of monoclonal antibodies for A*β*1-42, A*β*1-40, and A*β*1-38, and an additional fourth spot of streptavidin in each well. Plates were initially incubated with blocking buffer (5% BSA in phosphate buffered saline, 200 *μ*L per well) for 2 hours at ambient temperature and then with D2A6H-biotin conjugate (50 ng/mL in 1% BSA, phosphate buffered saline, 25 *μ*L per well) for 1 hour. Plates were then rinsed with phosphate buffered saline before addition of experimental samples for determination of A*β*1-42, A*β*1-40, A*β*1-38, and A*β*1-37 levels, following the manufacturer's instructions as for the A*β* 3-plex kit (catalog number K15148E-1). Rat brain extracts for use in the 4-plex assay were made in 0.2% diethylamine, as previously described [62]. For detection of rat A*β* peptides in the 4-plex (Figure 5), 252Q6-sulfo-tag conjugate was used, and for detection of human A*β* peptides from cell cultures 6E10-sulfo-tag conjugate was used. For A*β* in transiently transfected PS1/PS2 dKO fibroblasts, A*β*1-42 was quantified using an ELISA kit (WACO), and A*β*1-40 was quantified by ELISA as described above for H4-APPsw cultures. For triple transgenic mice (3xTg; [63]), human transgenic A*β*1-42 was assayed in brain homogenates using an ELISA kit (WACO). For the rat and mouse experiments illustrated in Figures 7 and 8, brain samples were prepared by solid phase extraction [64], and endogenous rat brain A*β*1-42 and A*β*1-40 were quantified by ELISA as previously described for wild type mice [65]. For brain extracts made using solid phase extraction, calibration of A*β* was relative, based on the approximately linear response of the assay in the range tested. For brain extracts made in 0.2% diethylamine, A*β*1-42, A*β*1-40, A*β*1-38, and A*β*1-37 concentrations in brain and cell culture samples were determined by fitting the results of immunoassays against calibration curves derived from a range of dilutions of the corresponding synthetic peptides on each assay plate using a quadratic curve fit (Graphpad Prizm 5.0). A*β*1-*x* was calibrated in the same way against synthetic A*β*1-40 peptide. Results were expressed in units of pM, corrected for sample dilution.

### 2.5. Immunodepletion of A*β*

For immunodepletion of rat brain A*β*, solid phase extracts [64] were pooled within treatment groups. The pools were divided into equal aliquots and incubated with or without monoclonals 565, TSD, 4G8, or 6E10 (10 *μ*g) at 4°C overnight. Protein G beads (50 *μ*L; EZview, Sigma) were added, and incubation was continued with agitation for 1 hour. The beads were removed by centrifugation, and A*β*1-40 and A*β*1-42 in the unbound fraction were quantified by ELISA, as described above.

### 2.6. Western Blotting

For western blotting of A*β* peptides from H4-APPsw cell cultures, A*β* was immunoprecipitated directly from the cell culture medium and was eluted from the protein G beads by addition of lithium dodecyl sulfate (LDS) electrophoresis sample buffer (Invitrogen). A*β* peptides were separated by gel electrophoresis in the presence of 8 M urea [66], transferred to PVDF membrane, and detected by western blotting using monoclonal 26D6-HRP conjugate.

For western blotting of APP-CTF in cell lysates, H4-APPsw cell cultures in T-75 flasks were rinsed with DPBS, harvested, isolated by centrifugation, and stored at −80°C until needed. Cells were suspended in SDS sample buffer (20,000 cells/*μ*L), boiled for 10 min, and centrifuged 3000 ×g for 5 min. Total protein content was determined using an assay kit (EZQ, Invitrogen cat number R33200). Proteins were separated by electrophoresis on Bis-Tris 16% polyacrylamide gels and transferred to nitrocellulose filters. APP-CTF*β* was detected using 26D6-HRP, and APP-CTF*α* was detected using ct695 polyclonal (Invitrogen, cat number 51-2700) and secondary goat anti-rabbit horseradish peroxidase conjugate (Zymed, catalog number 62-6120). Chemiluminescence images were captured and quantified using an imaging station (Fuji model number LAS-3000). The ct695 western blots also show APP-CTF*β*, which migrates as a fainter band above APP-CTF*α* under these conditions. To confirm the consistency of sample loading, the APP-CTF western blots were reprobed for glyceraldehyde-3-phosphate dehydrogenase (GAPDH) using monoclonal 1D4 (Enzo Life Sciences, cat number CSA-335).

For immunoprecipitation and western blotting of APP-CTF*β* and APP-CTF*α* from rat brain, weighed sagittal brain halves were homogenized using a rotary homogenizer (Polytron) in 5 volumes of RIPA buffer (Sigma R-0278; 150 mM NaCl, 1.0% IGEPAL CA-630, 0.5% sodium deoxycholate, 0.1% SDS, 50 mM Tris, pH 8.0) containing protease inhibitors (Roche complete cat number 11836145001) and centrifuged at 25,000 ×g for 30 min. All steps were carried out on ice or at 4°C. The pellet from centrifugation was discarded. Total protein concentration in the supernatant was determined using BCA protein assay (Pierce number 23227), and all samples were adjusted to a concentration of 18 mg/mL by addition of RIPA buffer. For APP-CTF*β* immunoprecipitation, 5 *μ*g of 252Q6 was added to 1 mL of homogenate and incubated on ice for 1 hour, and then 50 *μ*L of magnetic protein A/G beads (Thermo Scientific cat number 88803) was added, and incubation was continued overnight with mixing. Using a magnet to isolate the beads, beads were washed once with 1 mL RIPA and twice with 1 mL of Tris saline pH 7.5 (50 mM Tris pH 7.5, 15 mM NaCl). The beads were resuspended in 40 *μ*L of SDS sample buffer, boiled 5 mins, and removed using the magnet before gel electrophoresis and western blotting using ct695 polyclonal, as described above. For APP-CTF*α*, 5 *μ*g of 4G8 was added to homogenates previously used for immunoprecipitation of APP-CTF*β*, and immunoprecipitation using magnetic beads followed by western blotting using ct695 polyclonal was carried out as described above.

### 2.7. Mass Spectrometry of A*β* Peptides from Cell Cultures

H4-APPsw cell cultures were grown in T-75 flasks until 75% confluent and rinsed with Dulbecco's phosphate buffered saline (DPBS; Gibco cat number 14109), and 20 mL of DMEM supplemented with L-glutamine (2 mM), geneticin, penicillin, streptomycin, and DMSO with or without BMS-869780 was added (final concentration of DMSO was 0.2%). After incubation for 24 hours at 37°C in 5% CO_2_, culture medium was removed, centrifuged to remove cells, and frozen in aliquots at −80°C. For immunoprecipitation, aliquots of cell medium (4 mL) were thawed, followed by the addition of protease inhibitor cocktail (Sigma P-8340) to a final concentration of 1%, 60 ng of [^15^N]-A*β*1-40 synthetic peptide (rPeptide, 1101-1, Athens, GA), 30 *μ*g of monoclonal 26D6, and 15 *μ*g of monoclonal 4G8. After incubation for 20 min on ice, 80 *μ*L of protein G agarose beads (Pierce Rockford, IL) was added, and incubation was continued overnight at 4°C. Beads were isolated by centrifugation and washed three times by centrifugation in 1 mL of ice cold phosphate buffered saline and then washed a final time in 1 mL 10 mM Tris-HCl pH 8.0. A*β* was eluted from the beads using 30 *μ*L of 70% acetonitrile/0.1% formic acid. MALDI-TOF MS analysis was conducted using a Bruker Ultraflex III TOF/TOF mass spectrometer (Billerica, MA). A mix of A*β* standard peptides (AnaSpec, Fremont, CA) A*β*1-37 ([M+H]^+^ =* m/z* 4071.5), A*β*1-38 ([M+H]^+^ =* m/z* 4128.5), A*β*1-39 ([M+H]^+^ =* m/z* 4227.7), A*β*1-40 ([M+H]^+^ =* m/z* 4326.9), A*β*1-42 ([M+H]^+^ =* m/z* 4511.2), and [^15^N]A*β*1-40 ([M+H]^+^ =* m/z* 4378.4) was prepared to a final concentration of 1 ng/*μ*L in 50% : 50% acetonitrile : water (v : v). Both the standard peptide sample and the samples from H4-APPsw cell medium following immunoprecipitation (IP) were further processed by mixing 5 *μ*L sample with 5 *μ*L of 7 mg/mL MALDI matrix (*α*-cyano-4-hydroxycinnamic acid, CHCA, from Sigma-Aldrich, St. Louis, MO) in 70% : 30% acetonitrile : water (v : v) with 0.1% trifluoroacetic acid (v : v). 0.5 *μ*L of sample was spotted to an Anchorchip 384-well target plate (Bruker Daltonics, Billerica, MA) and allowed to dry in air before analysis. Analysis of various A*β* peptide isoforms from the standard peptides and the cell samples was performed in positive linear mode, accumulating 2000 spectra. Intensities of each analyte were normalized against a MALDI matrix peak (*m/z* 824.8) and the internal standard peak ([^15^N]A*β*1-40,* m/z* 4378.4).

### 2.8. Notch Signaling and Processing Assays

Inhibition of Notch signaling in cultured cells using a mouse-derived truncated Notch1 transgene, mNotch-ΔE [67], has been described in detail previously [59]. Western blots for mNotchΔ1865 and NICD using 9E10 anti-c-myc monoclonal were carried out as previously described [68].

### 2.9. Animals and Dosing

All experimental procedures with animals followed National Institutes of Health guidelines and were authorized by and in compliance with policies of the Bristol-Myers Squibb Animal Use and Care Committee. Mice and rats were housed with a 6:00 AM to 6:00 PM light/dark cycle and allowed free access to food and water. For 3xTg mice [63], BMS-869780 was dosed in three-month-old females by oral gavage at 6 mL/kg in vehicle consisting of 84% polyethylene glycol average molecular weight of 400 (PEG-400), 15% EtOH, and 1% Tween-80 (w/w/w). The compound was dissolved in EtOH and then diluted with PEG-400 and Tween, after which the vial was sealed, vortexed, and sonicated at 56°C for 1 hour. Animals were euthanized by asphyxiation in CO_2_. Blood was collected by cardiac puncture and placed into ethylene-diaminetetraacetic acid microtainer tubes for the preparation of plasma. The cerebellum was collected for the determination of compound concentration, and the remaining brain was separated into left and right halves before freezing in liquid nitrogen. For the rat time course study, 8- to 12-week-old female Sprague-Dawley rats were obtained from Charles River Laboratories (Wilmington, MA), BMS-869780 was dosed orally at 4 mL/kg, and brain and plasma samples were collected as described for the 3xTg mice. For the four day repeat dose rat study, BMS-869780 was given by oral gavage in vehicle consisting of PEG-400, PEG-200, D-*α*-tocopheryl polyethylene glycol succinate, Solutol HS 15, in the ratio 80 : 10 : 5 : 5 (w/w/w/w). Methods for the pharmacokinetics studies in rat, dog, and monkey are described below.

### 2.10. Pharmacokinetic to Pharmacodynamic (PK/PD) Relationship in Rodents

The data generated in the mouse and rat time course experiments were analyzed sequentially by nonlinear regression (WinNonlin Pharsight Corporation, Mountain View, CA). The pharmacokinetic data were best described by a 1-compartment linear model with first-order absorption and elimination. Subsequently, the pharmacodynamic parameters were estimated from the BMS-869780 plasma concentrations and the observed reductions of brain A*β*1-42 or A*β*1-40 by fitting to the equation for inhibition of synthesis [69]. The goodness-of-fit was determined by visual inspection, Akaike Information Criterion, Schwartz Criterion, examination of the residuals and the coefficient of variation of the parameter estimates.

### 2.11. Pharmacokinetics

Male Sprague-Dawley (SD) rats (300–350 g) were fasted overnight, and three per group received BMS-869780 either as an intravenous (IV) infusion in PEG400 : ethanol (90 : 10 w/w) at 1 mg/kg over 5 min via the jugular vein or as nanosuspension (d_50_ ca. 300 nm) by oral gavage at 5 mg/kg in Povidone K-30 : sodium lauryl sulfate : water (2.5 : 0.12 : 97.38 w/w/w/w). For the IV infusion, serial blood samples were obtained before dose and at 0.17, 0.25, 0.5, 0.75, 1, 2, 4, 6, 8, and 24 hours after dose. For the PO nanosuspension, serial blood samples were obtained before dose and at 0.25, 0.5, 0.75, 1, 2, 4, 6, 8, and 24 h after dose. Blood samples, ~0.3 mL, for all studies were collected from the jugular vein into K_3_EDTA-containing tubes and then centrifuged at 4°C (1500–2000 ×g) to obtain plasma, which was stored at −20°C until analysis by LC/MS/MS. In male beagle dogs, the PK of BMS-869780 was evaluated in a cross-over study design with a one-week washout between treatments. Dogs were fasted overnight, and three animals (9.5 to 10.7 kg) received BMS-869780 by IV infusion at 1 mg/kg over 5 minutes in PEG400 : ethanol (90 : 10 w/w) or as nanosuspension (d_50_ ca. 300 nm) by oral gavage at 5 mg/kg in Povidone K-30 : sodium lauryl sulfate : water (2.5 : 0.12 : 97.38 w/w/w/w). Serial blood samples (~0.3 mL) were collected from a saphenous vein into K_3_EDTA-containing tubes before dose and at 0.083, 0.17, 0.25, 0.5, 0.75, 1, 2, 4, 6, 8, and 24 hours after IV dose, and 0.25, 0.5, 0.75, 1, 2, 4, 6, 8, and 24 hours after oral dose, followed by centrifugation at 4°C (1500 to 2000 ×g) to obtain plasma. Samples were stored at −20°C until analysis of BMS-869780 levels by LC-MS/MS. In male cynomolgus monkeys, the PK of BMS-869780 was evaluated in a cross-over study design with a 1-week washout between treatments. Following an overnight fast, three animals (4.5 to 8 kg) received BMS-869780 by IV infusion via a femoral vein at 1 mg/kg over 10 minutes in PEG400 : ethanol (90 : 10), or by oral gavage at 5 mg/kg in Povidone K-30 : sodium lauryl sulfate : water (2.5 : 0.12 : 97.38). Serial blood samples, ~0.3 mL, were collected from a femoral artery into K_3_EDTA-containing tubes before dose and at 0.083, 0.17, 0.25, 0.5, 0.75, 1, 2, 4, 6, 8, and 24 hours after IV dose, and 0.25, 0.5, 0.75, 1, 2, 4, 6, 8, and 24 hours after oral dose, followed by centrifugation at 4°C (1500 to 2000 ×g) to obtain plasma. Samples were stored at −20°C until analysis of BMS-869780 levels by LC-MS/MS. The PK parameters of BMS-869780 were obtained by noncompartmental analysis of plasma concentration versus time data (WinNonlin software, Version 5.0; Pharsight Corporation, Mountain View, CA). The peak concentration (*C*_max⁡_) and time for *C*_max⁡_ (*T*_max⁡_) were recorded directly from experimental observations. The area under the curve from time zero to the last sampling time (AUC^0-*T*^) and the area under the curve from time zero to infinity (AUC^INF^) were calculated using a combination of linear and log trapezoidal summations. The total plasma clearance (CLTp), steady-state volume of distribution (Vss), apparent elimination half-life (*T*_1/2_), and mean residence time (MRT) were estimated after IV administration. The absolute oral bioavailability (*F*) was estimated as the ratio of dose-normalized AUC values following oral and IV doses.

### 2.12. Prediction of Human Dose

Interspecies allometric scaling adjusted for brain weight was used to predict human CLTp [70], while simple allometric scaling was used to predict Vss [71]. Briefly, the Vss and brain weight adjusted CLTp values from nonclinical species were plotted against body weight on a log-log scale to yield estimates of Vss and CLTp × brain weight in humans. The estimated CLTp × brain weight for human was adjusted for the brain weight of humans to yield a predicted CLTp. All of the nonclinical species demonstrated mono- or biexponential plasma concentration-time profiles; therefore, the MRT method was used to simulate the human pharmacokinetic profile as described below. Noncompartmental analysis was performed using WinNonlin software (version 5.0; Pharsight Corporation, Mountain View, CA). The *k*_a_ for each species was obtained from the IV and PO (nanosuspensions) of rat, dog, and cynomolgus monkey by deconvolution of plasma concentration-time data using Kinetica (version 5.0; Seattle, WA). The bioavailability was estimated from the nanosuspensions from rat, dog, and cynomolgus monkeys. The average *k*_a_ and* F*, along with the Vss and CLTp from allometric scaling, were incorporated into a two-compartment model to predict the human oral plasma concentration-time profiles. Human steady state doses to achieve plasma AUCs comparable to those in rats which produced 25% ABEC (area between baseline and A*β* effect-time curve) reductions were estimated. The results are summarized in Tables 3 and 4.

### 2.13. Determination of BMS-869780 Concentrations

Plasma and brain samples were analyzed using an ultraperformance liquid chromatography-tandem mass spectrometry (UPLC-MS-MS) method. The UPLC MS-MS system consisted of a Waters Aquity Ultra Performance LC Sample Organizer, Solvent Manager and Sample Manager, a Waters BEH C18, 1.7 u 50 × 2.1 mm, column operated at 60°C, and a SCIEX API 4000 Q trap mass spectrometer. The mobile phase consisted of (A) water with 0.1% formic acid and (B) acetonitrile with 0.1% formic acid, delivered at 600 *μ*L/min using a gradient program. The initial elution condition was 5% B which was maintained for 0.2 min and increased to 95% B in 0.5 min and maintained for 0.4 min. It was then returned to 5% B in 0.1 min and maintained for 0.2 min. The MS-MS analysis was performed using the heated nebulizer under positive ion mode with the source temperature at 400°C. The capillary voltage was 5000 eV and the collision energy 49 eV. The mass-to-charge ratios of 453 (precursor ion) and 438 (product ion) were used for multiple reaction mode monitoring of BMS-869780. The quantitation range for BMS-869780 was 1 to 5000 nM. Plasma samples were deproteinized and extracted with four portions of acetonitrile. Brain samples (0.1 g) were homogenized in 0.4 mL of acetonitrile.

### 2.14. Other Methods

Determination of IC_50_ values for inhibition of secreted alkaline phosphatase (SEAP) was carried out using an H4 cell line stably expressing SEAP. Cells were treated overnight with compounds in 384-well format, and SEAP accumulation in the culture media was quantified using a chemiluminescence substrate. The pregnane-X-receptor transactivation assay (PXR-TA) was based on the methods of Goodwin et al. [72] as described previously [59]. In the 4-day rat study with BMS-869780, tissues were fixed in 10% neutral buffered formalin, embedded in paraffin, sectioned, stained with hematoxylin and eosin, and examined by light microscopy.

## 3. Results

### 3.1. Identification of BMS-869780

The first compounds reported to have GSM activity were NSAIDs that exhibited selective inhibition of A*β*1-42 production [28]. A high throughput screen (HTS) was therefore carried out using FRET-based immunoassays for A*β*1-42 and A*β*1-40 levels in H4-APPsw cultures. Approximately 10^6^ compound samples were incubated for 24 hours in H4-APPsw cell cultures, one compound per well in 384-well format, at a single concentration of 13 *μ*M. The 22,304 samples exhibiting greater than 50% inhibition of A*β*1-42 were subsequently retested in triplicate, that is, three additional wells at 13 *μ*M. This yielded 10,010 samples for which the average inhibition of A*β*1-42 in the four test wells was greater than 50%. After elimination of some samples due to previously known A*β* inhibition or chemical reactivity, 8,013 compounds were organized into clusters of related structures based on structural similarities [73], and 2,013 representative compounds were chosen for further evaluation. To rule out nonspecific effects on production or secretion of A*β*, dose response curves were determined for secreted alkaline phosphatase (SEAP) and for A*β*1-42 to compare the IC_50_ values. This yielded 409 samples that were relatively selective for A*β*1-42 (using a cutoff of ≥5-fold SEAP/A*β*1-42 IC_50_ ratio). To assess possible selectivity for A*β*1-42 lowering, IC_50_ values were determined in parallel for A*β*1-42 and A*β*1-40 in ca. 400 samples, and a series of compounds was identified from which BMS-869780 was subsequently derived through iterative improvements in potency and off-target profiles [54, 55]. An outline of the screening tiers and results obtained is shown in Figure 1(b), and the principle of the A*β* assays is shown in Figures 1(c) and 1(d).

### 3.2. Potency of BMS-869780 and Related Compounds for A*β*1-42 and A*β*1-40-Lowering

**Figure 2:**
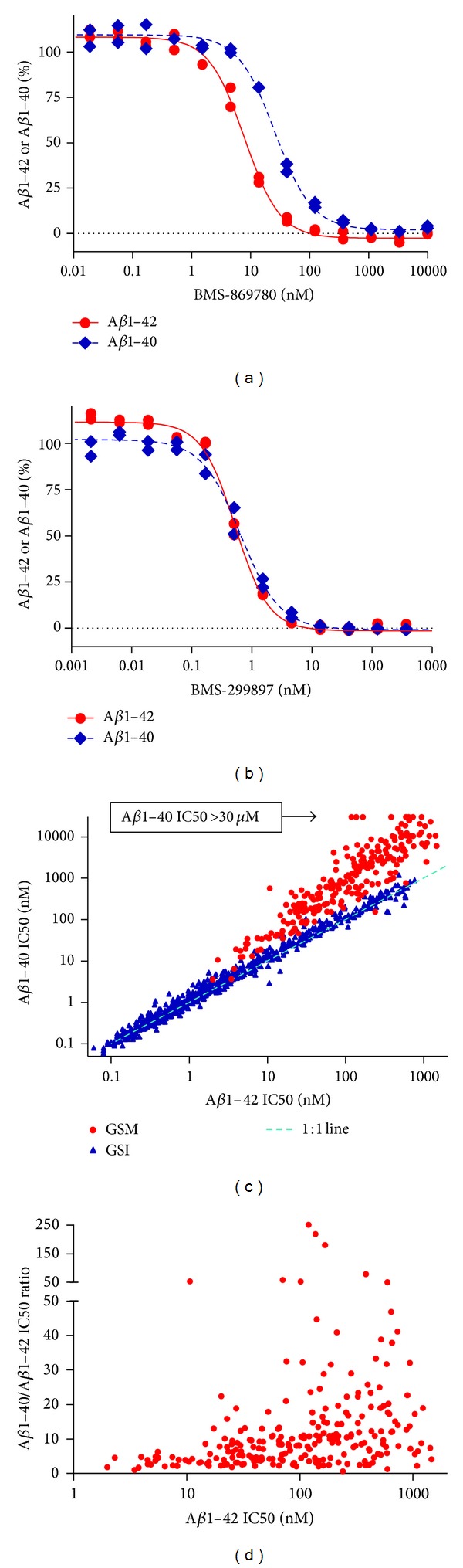
*In vitro* potency of BMS-869780 in the HTS assay. (a) H4-APPsw cultures were treated overnight with BMS-869780 (a GSM) at a range of concentrations and the relative levels of A*β*1-42 (red ●) and A*β*1-40 (blue ♦) were determined for calculation of IC_50_ values (summarized in Table 1). (b) H4-APPsw cultures were treated overnight with BMS-299897 (a representative GSI) as described for BMS-869780 in panel (a). IC_50_ values are summarized in Table 1. (c) IC_50_ values for A*β*1-42 and A*β*1-40 lowering were determined for 236 GSM (red ●) and for 688 GSI that were mostly of the type containing the aryl sulfonamide core (blue ▲). For some compounds, the A*β*1-40 IC_50_ value was greater than 30 *μ*M, the highest concentration tested in the A*β*1-40 assay (arrow). (d) The ratio of the A*β*1-40 IC_50_ to A*β*1-42 IC_50_ was plotted against A*β*1-42 IC_50_ for the same 236 GSM illustrated in (c).

While many of the ca. 400 samples showed wide separations between the A*β*1-42 and A*β*1-40 IC_50_ values, BMS-869780 itself showed only a four-fold separation between the IC_50_ values (Figure 2(a)), presenting a minimal contrast with GSIs such as BMS-299897 (Figure 2(b)) and BMS-433796 (see summary of IC_50_ values in Table 1). Nevertheless, as a group, compounds chemically related to BMS-869780 showed limited overlap with GSIs based on the separation of A*β*1-42 and A*β*1-40 IC_50_ values, as illustrated for 236 GSMs and 688 GSIs (Figure 2(c)). This implies different A*β*-lowering mechanisms between the two groups. The ratios between A*β*1-42 and A*β*1-40 IC_50_ values in the GSM group ranged from little more than two-fold to almost 250-fold, with a trend toward lower ratios for compounds with lower IC_50_ values (Figure 2(d)). Anticipating subsequent experiments in the 3xTg mouse, the effect of the presenilin M146V FAD mutant on BMS-869780 potency was evaluated. MEF cell cultures lacking endogenous presenilins were therefore cotransfected with human APP-CTF*β* and human presenilin, either wild type or M146V allele. The IC_50_ for A*β*1-42 was ca. 3-fold higher in cultures expressing the M146V allele, relative to cells expressing wild type presenilin. Likewise, A*β*1-40 IC_50_ values were ca. 3-fold shifted, although the IC_50_ appeared lower for A*β*1-40 in the wild type MEF cell cultures than in the H4-APPsw cultures. Thus, BMS-869780 appeared to be a little less potent in the context of the presenilin-1 M146V allele. IC_50_ values are summarized in Table 1.

### 3.3. Evidence for the Noninhibitory Mechanism of BMS-869780* In Vitro*

**Figure 3:**
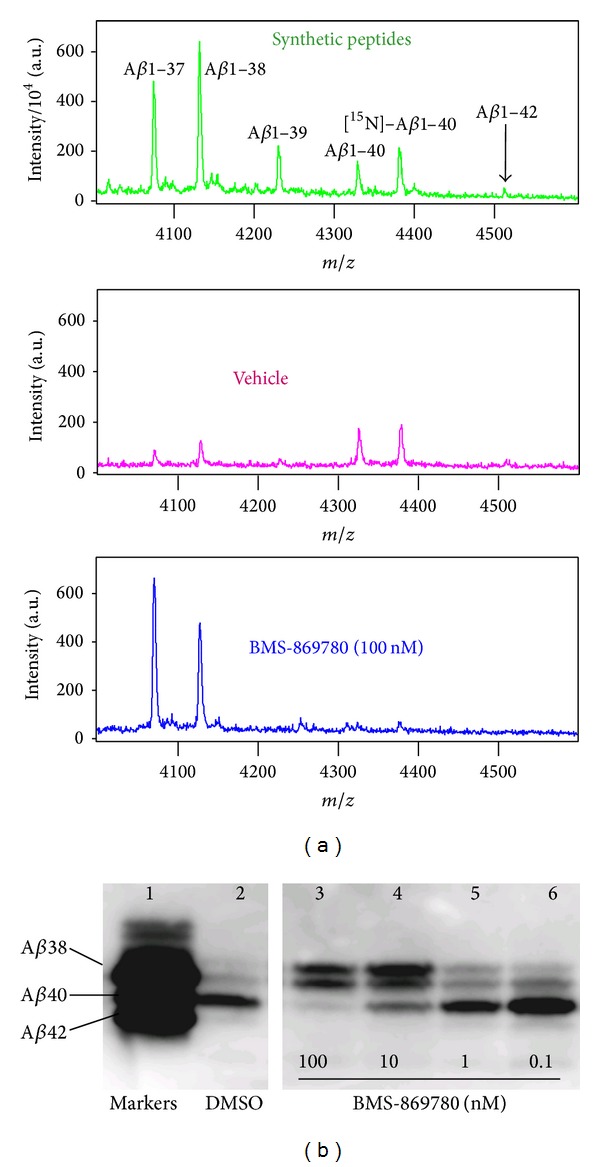
BMS-869780 increased the levels of the shorter peptides A*β*1-38 and A*β*1-37. (a) Top panel: an equimolar mix of synthetic peptides A*β*1-37, A*β*1-38, A*β*1-39, A*β*1-40, [^15^N]-A*β*1-40, and A*β*1-42 was evaluated by MALDI-TOF mass spectrometry. H4-APPsw cell cultures were treated with vehicle (0.1% DMSO—middle panel) or BMS-869780 (100 nM—bottom panel), [^15^N]-A*β*1-40 was added, and A*β* peptides were immunoprecipitated and evaluated by MALDI-TOF mass spectrometry. (b) H4-APPsw cell cultures were treated with BMS-869780 or DMSO vehicle. A*β* peptides were separated by gel electrophoresis in the presence of urea and detected by western blotting. Under these conditions, higher molecular weight A*β* peptides exhibit greater gel mobility. Lane 1: an equimolar mix of synthetic peptides A*β*1-38, A*β*1-40, and A*β*1-42. Lane 2: DMSO vehicle-treated cell culture. Lanes 3–6: Cell cultures treated with BMS-869780 at final concentrations of 100, 10, 1 and 0.1 nM, respectively.

**Figure 4:**
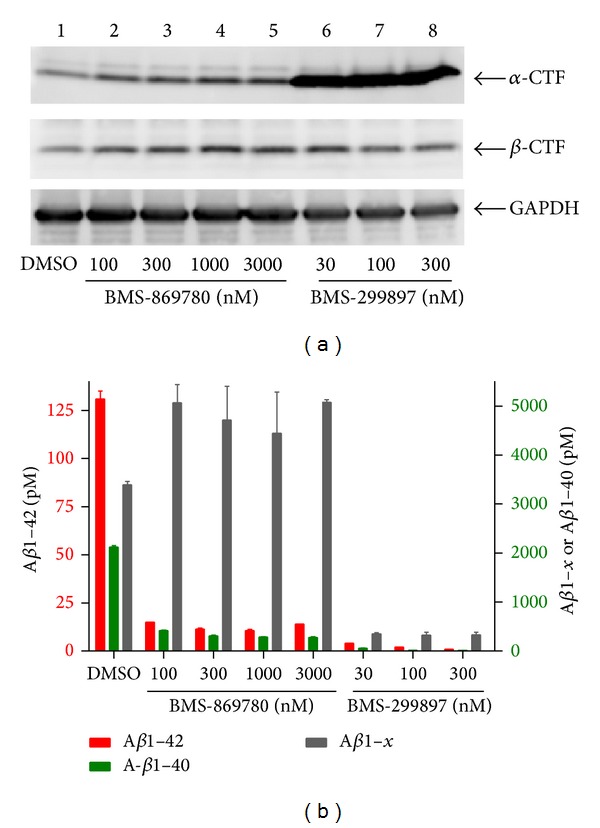
BMS-869780 had minimal effect on APP-CTF accumulation* in vitro*. H4-APPsw cell cultures were treated overnight with the indicated concentrations of BMS-869780, BMS-299897 or vehicle (0.1% DMSO). (a) Cells were harvested and analyzed by western blotting for APP-CTF*α*, APP-CTF*β*, and GAPDH. Lane 1; culture treated with vehicle 0.1% DMSO. Lanes 2–5; cultures treated with BMS-869780 at 100 nM, 300 nM, 1000 nM, or 3000 nM, respectively. Lanes 6–8; cultures treated with BMS-299897 at 30 nM, 100 nM, or 300 nM, respectively. (b) Levels of A*β*1-42 (red; left *Y*-axis), A*β*1-40 (green; right *Y*-axis), and A*β*1-*x* (grey; right *Y*-axis) were quantified.

**Figure 5:**
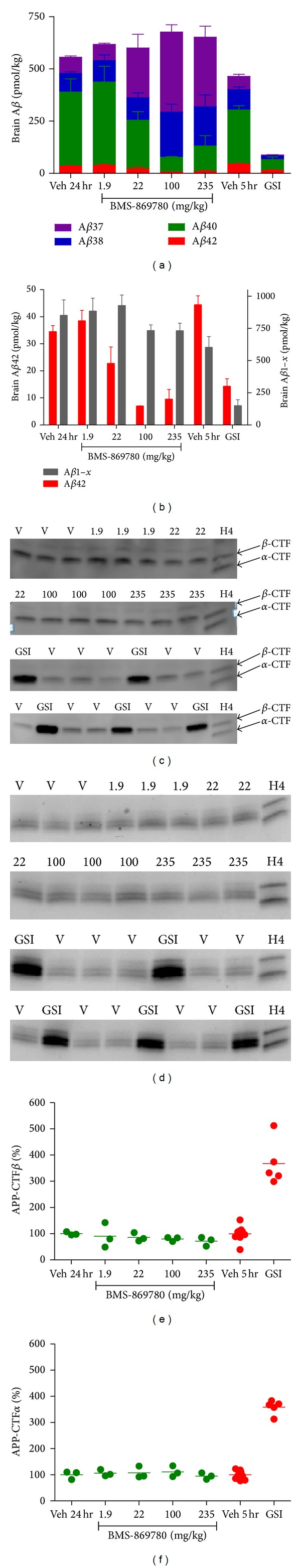
BMS-869780 modulated A*β* but did not cause accumulation of *β*CTF or *α*CTF in rat brain. Rats were given oral doses of BMS-869780, and levels of brain A*β*, *β*CTF, and *α*CTF were determined 24 hours later. For comparison, BMS-698861 was dosed in a separate experiment and samples were taken 5 hours later. (a) Brain levels of A*β*1-42 (red), A*β*1-40 (green), A*β*1-38 (blue), and A*β*1-37 (purple) are shown as bars stacked upon one another. The total height of each bar therefore represents the sum of the four peptides. (b) A*β*1-42 (red—left* Y* axis) and A*β*1-*x* (grey—right* Y* axis). The same results for A*β*1-42 are plotted in both (a) and (b). (c) Rat brain *β*CTF was detected by western blotting of immunoprecipitates from samples of the same rat brains used for A*β* determinations. V, vehicle groups; results from rats dosed with 1.9, 22, 100, and 235 mg/kg of BMS-869780 and 10 mg/kg BMS-698861 (GSI) are indicated. (d) Western blots of immunoprecipitated *α*CTF from the same rat brain samples. (e) and (f) quantification of western blots shown in (c) and (d), respectively, expressed relative to percent of average level of CTF in vehicle-treated rats. Actual doses of BMS-869780 were determined by analysis of concentrations in left-over dosing solutions.

BMS-869780 showed only a 4-fold shift in IC_50_ values between A*β*1-42 and A*β*1-40. The effect of BMS-869780 on A*β*1-37 and A*β*1-38 was therefore evaluated, as a more diagnostic test of the GSM mechanism [25]. H4-APPsw cultures were treated overnight with BMS-869780, and A*β* peptides were evaluated by mass spectrometry and western blotting. Using MALDI mass spectrometry, A*β*1-37, A*β*1-38, and A*β*1-40 were readily detected in vehicle-treated cultures, although A*β*1-42 levels were near the limit of quantitation. After treatment with BMS-869780 (100 nM), A*β*1-37 and A*β*1-38 were dramatically increased, whereas A*β*1-40 and A*β*1-42 were essentially undetectable (Figure 3(a)). The same conclusion was reached in experiments using a western blot method that separates different forms of A*β*. Increased levels of the shorter A*β*1-37 and A*β*1-38 peptides, which migrate more slowly than A*β*1-40 or A*β*1-42 by this method, were observed in cultures treated with BMS-869780 at 100 nM and 10 nM (Figure 3(b), lanes 3 and 4, resp.). Thus, taking the experimental data illustrated in Figures 2 and 3 together, BMS-869780 increased A*β*1-37 and A*β*1-38, while decreasing A*β*1-40 and A*β*1-42. This suggested that BMS-869780 would have a minimal effect, if any, on APP-CTF*α* and APP-CTF*β* turnover. H4-APPsw cell cultures were therefore treated at high concentrations, relative to the IC_50_s, of BMS-869780 and the GSI BMS-299897. For APP-CTF*α*, BMS-299897 caused an 8-fold increase in APP-CTF*α*, whereas BMS-869780 showed a 1.4-fold increase, averaged across doses (Figure 4(a)). The two compounds also showed a dramatic contrast in their effects on A*β* under these conditions. Whereas the GSI BMS-299897 dramatically reduced all A*β*1-*x* peptides, including A*β*1-42 and A*β*1-40, BMS-869780 selectively lowered A*β*1-42 and A*β*1-40, without any decrease in the overall levels of A*β*1-*x* (Figure 4(b)). In contrast to the result for APP-CTF*α*, APP-CTF*β* levels were not affected in this experiment by either compound (Figure 4(a)), suggesting that *γ*-secretase was not a major pathway for APP-CTF*β* turnover under these conditions. Indeed, it was recently reported that APP-CTF*β* turnover in H4 cells occurs largely through proteasomal and lysosomal pathways, in contrast to APP-CTF*α* turnover which is more dependent on *γ*-secretase [74]. Thus, the effect of BMS-869780 on APP-CTF*β* could not be directly evaluated in the H4-APPsw cell line under these conditions. To address the effect of BMS-869780 on APP-CTF*β*, experiments were carried out in the context of the intended target organ, that is, in the brain of rats given oral doses of BMS-869780. The GSI BMS-698861 was dosed for comparison. BMS-869780 decreased A*β*1-40 and A*β*1-42 and increased A*β*1-37 and A*β*1-38 in rat brain. The sum total of A*β*1-40, A*β*1-42, A*β*1-38, and A*β*1-37 suggested no significant change in overall A*β* levels (Figure 5(a)). This was consistent with results obtained in the A*β*1-*x* assay, which showed no significant decrease despite the robust decrease in A*β*1-42 (Figure 5(b)). In contrast, BMS-698861 decreased levels of all the peptides, A*β*1-40, A*β*1-42, A*β*1-38, A*β*1-37, and A*β*1-*x* (Figures 5(a) and 5(b)). APP-CTF*β* and APP-CTF*α* in samples of the same rat brains were evaluated by immunoprecipitation and western blotting. Neither APP-CTF*β* nor APP-CTF*α* levels were affected in rats given BMS-869780, whereas levels of both peptides increased several-fold in rats given the GSI BMS-698861 (Figures 5(c)–5(f)). Thus, in brain, inhibition of *γ*-secretase by BMS-698861 resulted in APP-CTF*β* and APP-CTF*α* accumulation, whereas modulation of A*β* by BMS-869780 had no effect on APP-CTF*β* or APP-CTF*α* levels.

### 3.4. BMS-869780 Does Not Inhibit Notch Processing

**Figure 6:**
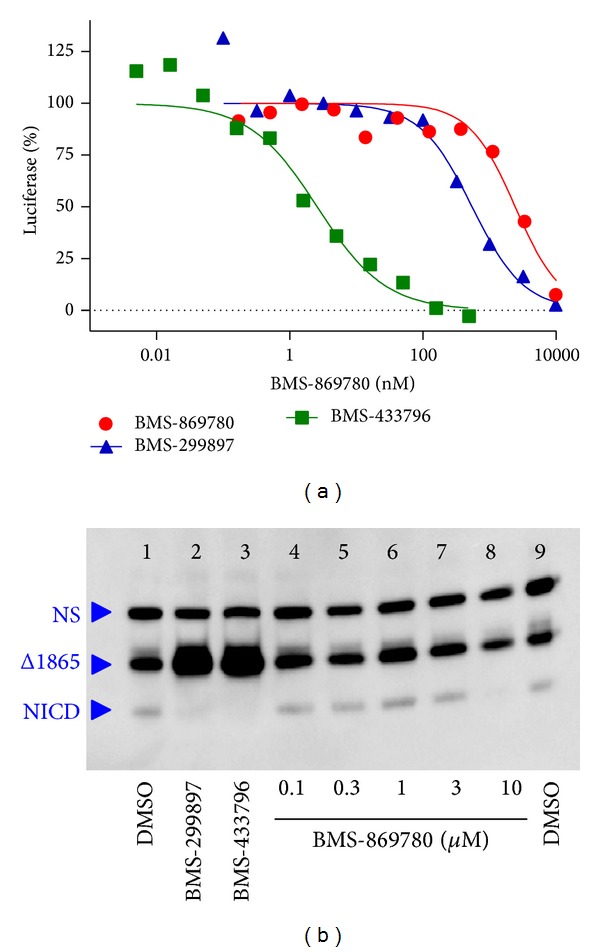
BMS-869780 did not inhibit Notch cleavage* in vitro*. (a) HeLa cell cultures were transfected with mNotchΔE and CBF1-luciferase reporter constructs, treated overnight with BMS-869780 (●), BMS-299897 (▲), or BMS-433796 (■), and luciferase assays were carried out. (b) HeLa cell cultures were transfected with mNotchΔ1865, treated with compounds overnight, and cell extracts were evaluated by western blot using anti-c-myc-HRP conjugate. Lanes 1 and 9: DMSO (0.1%) vehicle. Lane 2: BMS-299897 at 1 *μ*M. Lane 3: BMS-433796 at 0.3 *μ*M. Lanes 4–8: BMS-869780 at 0.1, 0.3, 1, 3, and 10 *μ*M, respectively.

The effect of BMS-869780 on Notch processing was evaluated using transcriptional reporter assays and western blotting of NICD levels. HeLa cell cultures were transfected with mNotch1ΔE and CBF1-luciferase reporter constructs and treated with BMS-869780. In most replicates of this experiment, inhibition of luciferase reporter occurred with IC_50_ > 10 *μ*M. In contrast, the GSIs BMS-299897 and BMS-433796 robustly inhibited luciferase activation, with IC_50_ = 340 nM and 2.1 nM, respectively (Figure 6(a)). The IC_50_ values for Notch-dependent luciferase expression are summarized in Table 1. To further evaluate the effect of BMS-869780 on Notch processing, western blots of cell cultures were carried out using mNotch1Δ1865, a truncated version of mNotch1ΔE that facilitates separation of NICD product from mNotch1 substrate on western blots [68]. HeLa cell cultures were transfected with mNotch1Δ1865 and treated overnight with compounds before western blotting. BMS-869780 had no effect on NICD levels at concentrations up to 3 *μ*M, whereas the GSIs, BMS-299897, and BMS-433796, greatly reduced NICD (Figure 6(b)). Furthermore, the GSIs caused an increased level of mNotch1Δ1865 substrate, most likely due to inhibition of its turnover by *γ*-secretase, as previously noted by Blat et al. [68]. In contrast, 10 *μ*M BMS-869780 did not increase mNotch1Δ1865 levels (Figure 6(b)), suggesting that the concomitant loss of NICD at 10 *μ*M was due to a nonspecific effect, rather than inhibition of mNotch1Δ1865 turnover. This was also consistent with the observation of detached and dead cells in the presence of 10 *μ*M BMS-869780.

### 3.5. Evaluation of BMS-869780 PK/PD and Residual Levels of A*β*

**Figure 7:**
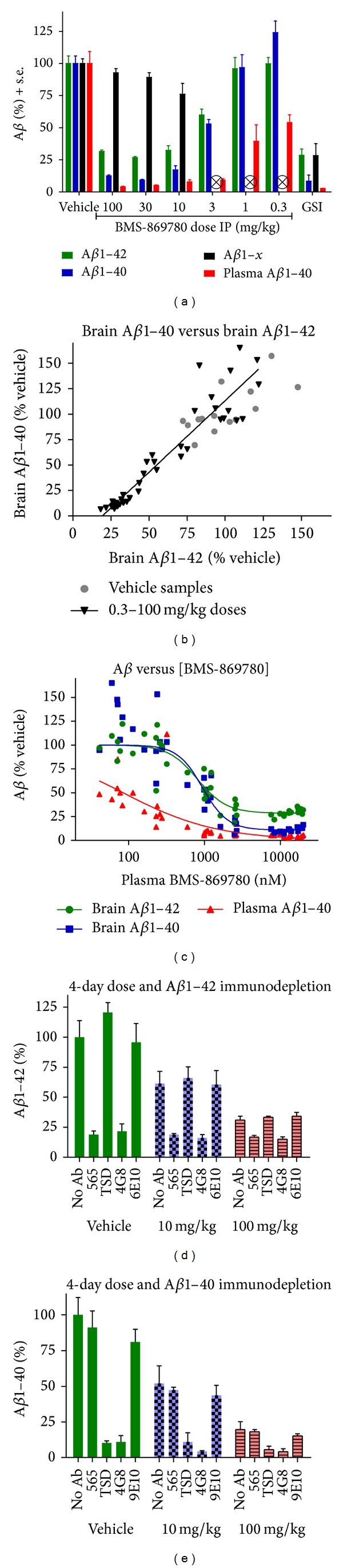
BMS-869780 dose response and evaluation of residual A*β* levels in rat brain. (a) Groups of rats received intraperitoneal (IP) injections of vehicle or BMS-869780 at doses of 100, 30, 10, 3, 1, and 0.3 mg/kg. Additional rats were dosed with GSI BMS-698861 at 30 mg/kg as a positive control for A*β*-lowering. Group sizes were seven rats for each dose of BMS-869780 and 14 rats for vehicle and GSI dose groups. Brain and plasma were harvested 5 hours after dosing. Brain A*β*1-42 (green), brain A*β*1-40 (blue), brain A*β*1-*x* (black), and plasma A*β*1-40 (red) were determined. Values are expressed as % relative to vehicle group mean. Whiskers represent standard error. ⊗A*β*1-*x* was not determined in the groups dosed at 3, 1, and 0.3 mg/kg. (b) Brain A*β*1-40 was plotted against brain A*β*1-42 for each rat dosed with BMS-869780 (black ▼) and for each rat dosed with vehicle (grey ●). Values are expressed as % relative to vehicle group mean. Whiskers represent standard error. (c) Brain A*β*1-42 (●), brain A*β*1-40 (■), and plasma A*β*1-40 (▲) were plotted against plasma concentration of BMS-869780 and the data were evaluated by fit to a four-parameter dose response curve. The top of the dose response curve was defined by vehicle group mean (100%), and the apparent IC_50_ values in terms of the plasma BMS-869780 concentration obtained for brain A*β*1-42, brain A*β*1-40, and plasma A*β*1-40 were 807 nM, 943 nM, and 84 nM, respectively. The respective 95% confidence intervals were 618–1053 nM, 704–1264 nM, and 44–158 nM. (d) Rats were dosed once daily with BMS-869780 for 4 days at 10 and 100 mg/kg or vehicle, plasma, and brain samples were taken 5 hours after the last dose, and immunodepletion of brain extracts was carried out prior to A*β*1-42 ELISA assays. Specific monoclonals used were 565 (A*β*1-42 selective), TSD (A*β*1-40 selective), 4G8 (binds both A*β*1-42 and A*β*1-40), and 6E10 (does not bind rat A*β*). After immunodepletion, A*β*1-42 was assayed by ELISA. (e) Same as described in (d), except that A*β*1-40 ELISA was carried out following the immunodepletion.

To evaluate BMS-869780 PK/PD, rats were given BMS-869780 intraperitoneally at a range of doses from 0.3 mg/kg to 100 mg/kg. The GSI BMS-698861 was dosed at 30 mg/kg for comparison. Five hours after dosing, brain A*β*1-42 and brain A*β*1-40 exhibited dose-dependent lowering with ca. 50% lowering at 3 mg/kg, and plasma A*β*1-40 lowering was dose dependent with ca. 50% lowering at 0.3 mg/kg (Figure 7(a)). In contrast, brain A*β*1-*x* levels were not affected by BMS-869780 (A*β*1-*x* was evaluated only in samples from the highest three doses). For comparison, the GSI, BMS-698861, caused robust lowering in all A*β* assays, including A*β*1-*x* (Figure 7(a)). The concentration of BMS-869780 in plasma associated with 50% lowering of brain A*β*1-42 was ca. 1 *μ*M (Figure 7(c)). A*β*1-42 appeared to be less completely inhibited than A*β*1-40. The linear correlation of A*β*1-42 with A*β*1-40 showed an intercept of 19.4% on the A*β*1-42 axis (Figure 7(b)), indicating a higher assay signal for A*β*1-42 relative to A*β*1-40 under conditions of maximal inhibition. Furthermore, at high plasma concentrations of BMS-869780, the best fit curve suggested a residual 29% A*β*1-42 and 11% A*β*1-40 assay signal remaining (Figure 7(c)). To investigate the residual assay signal further, brain A*β*1-42 and brain A*β*1-40 were evaluated in rats given four daily doses of BMS-869780 to achieve high sustained exposures, and residual levels of brain A*β*1-42 and A*β*1-40 were evaluated by immunodepletion. Despite the high exposures achieved (Table 4) and the extended time period in this experiment, residual ELISA signals for A*β*1-42 of 31% and for A*β*1-40 of 19% were observed (Figures 7(d) and 7(e)). Immunodepletion of the samples prior to ELISA was carried out to determine how much of the assay signal was due to residual A*β*1-42 or A*β*1-40. Specific monoclonals 565 and TSD selectively depleted A*β*1-42 and A*β*1-40, respectively, whereas monoclonal 4G8, which binds both, depleted both A*β*1-42 and A*β*1-40. The monoclonal 6E10, which selectively binds human A*β* relative to rat A*β*, depleted neither A*β*1-42 nor A*β*1-40. After immunodepletion, there was a residual signal of 16% for A*β*1-42 and 4% for A*β*1-40, averaged across depleted samples in both dose groups (Figures 7(d) and 7(e)). Thus, the residual signal appeared to be a combination of nonspecific background signal and small residual pools of ca. 15% A*β*1-42 and 7% A*β*1-40. This residual A*β*1-42 may represent either a slow turnover pool of A*β*1-42 or a source of A*β*1-42 production that is not readily inhibited by *γ*-secretase-targeted compounds.

**Figure 8:**
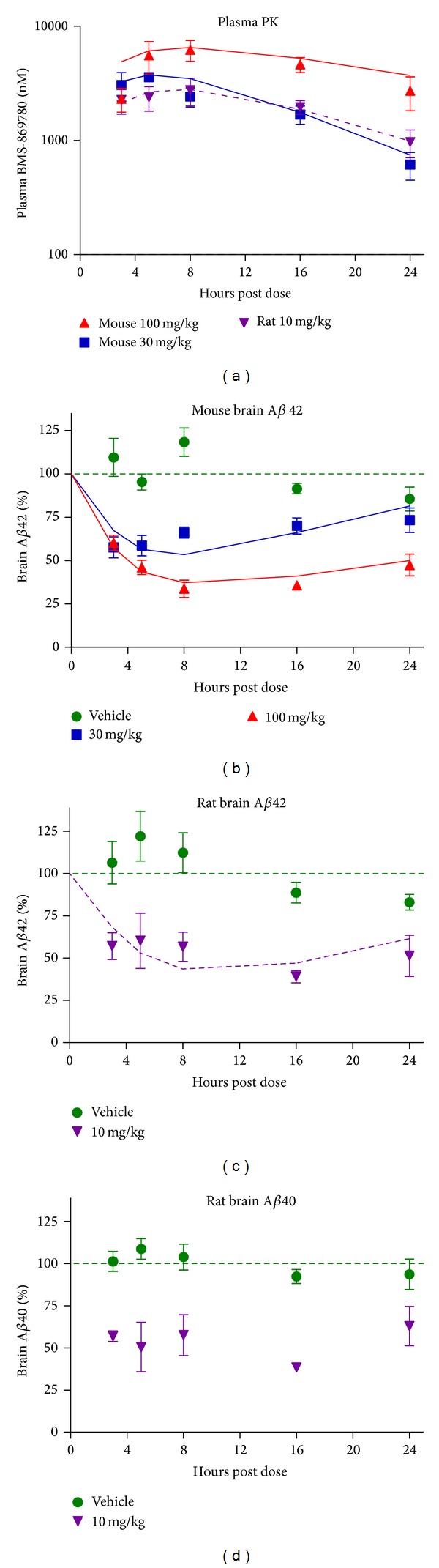
Analysis of the PK/PD relationship for BMS-869780 in rats and mice. Rats were dosed orally with BMS-869780 at 10 mg/kg, and triple transgenic mice were orally dosed at 30 mg/kg and 100 mg/kg. Additional groups of rats and mice were dosed with vehicle alone. Brain and plasma were harvested at 3, 5, 8, 16, and 24 hours after dosing for determination of brain A*β*1-42 and plasma BMS-869780 concentration. Group sizes were 5 rats or 4 mice. Whiskers represent standard error. (a) Plasma concentrations of BMS-869780 were determined for the mice dosed at 100 mg/kg (▲) and 30 mg/kg (■) and for the rats dosed at 10 mg/kg (▼). The data were fit to a one-compartment PK model and the predicted plasma BMS-869780 concentrations are shown for mouse (solid lines) and rat (broken line). (b) Brain A*β*1-42 levels were determined for the mice dosed with BMS-869780 at 100 mg/kg (▲), 30 mg/kg (■), or vehicle alone (●). The data were fitted using the indirect pharmacodynamic response model and predicted values are shown (solid lines). (c) Brain A*β*1-42 levels were determined for the rats dosed with BMS-869780 at 10 mg/kg (▼) or vehicle alone (●). The data were fitted using the indirect pharmacodynamic response model and predicted values are shown (dashed line). The values of the PK and PD parameters determined from these experiments are summarized in Table 2. (d) Brain A*β*1-40 levels determined in the same rats as illustrated in (c).

To evaluate the pharmacokinetic to pharmacodynamic (PK/PD) relationship for BMS-869780 in more detail, time courses of A*β* lowering and plasma BMS-869780 concentration were carried out in mice and rats. The PK and PK/PD were then modeled sequentially using 1-compartment PK and indirect response PK/PD models, respectively. Triple transgenic mice [63] were dosed orally at 30 mg/kg or 100 mg/kg, and brain and plasma were taken from groups of animals after 3, 5, 8, 16, and 24 hours. Rats were dosed orally at 10 mg/kg, and brain and plasma were taken at the same time points as for mice. Plasma BMS-869780 showed increasing concentration until 4–8 hours then decreased (Figure 8(a)). Brain A*β*1-42 in the mouse, and both A*β*1-42 and A*β*1-40 in the rat, decreased until 8–16 hours relative to vehicle-treated groups (Figures 8(b)–8(d)). The* in vivo* plasma IC_50_ values for brain A*β*1-42 were 1.9 *μ*M and 4.0 *μ*M for rat and mouse, respectively. After taking account of the protein bound fractions (99.5% and 99.6% in rat and mouse, resp.), these are within 2-fold factors of the* in vitro* IC_50_ values of 5.1 nM and 22 nM for wild type and presenilin-1 M146V alleles, respectively (Table 1). Estimates of the PK and PD parameters are summarized in Table 2. Using the PK and PK/PD models for the rat data, steady-state assuming linear PK was predicted to occur after three daily doses, and a 3 mg/kg oral dose of BMS-869780 was predicted to yield AUC^24 h^ = 18.6 *μ*M·h with corresponding A*β*1-42 ABEC^0-24 h^ = 26.9% at steady state. Observed and predicted values of dose, AUC, and ABEC in rat and mouse are summarized in Table 3.

### 3.6. Pharmacokinetics and Human Dose Prediction

**Figure 9:**
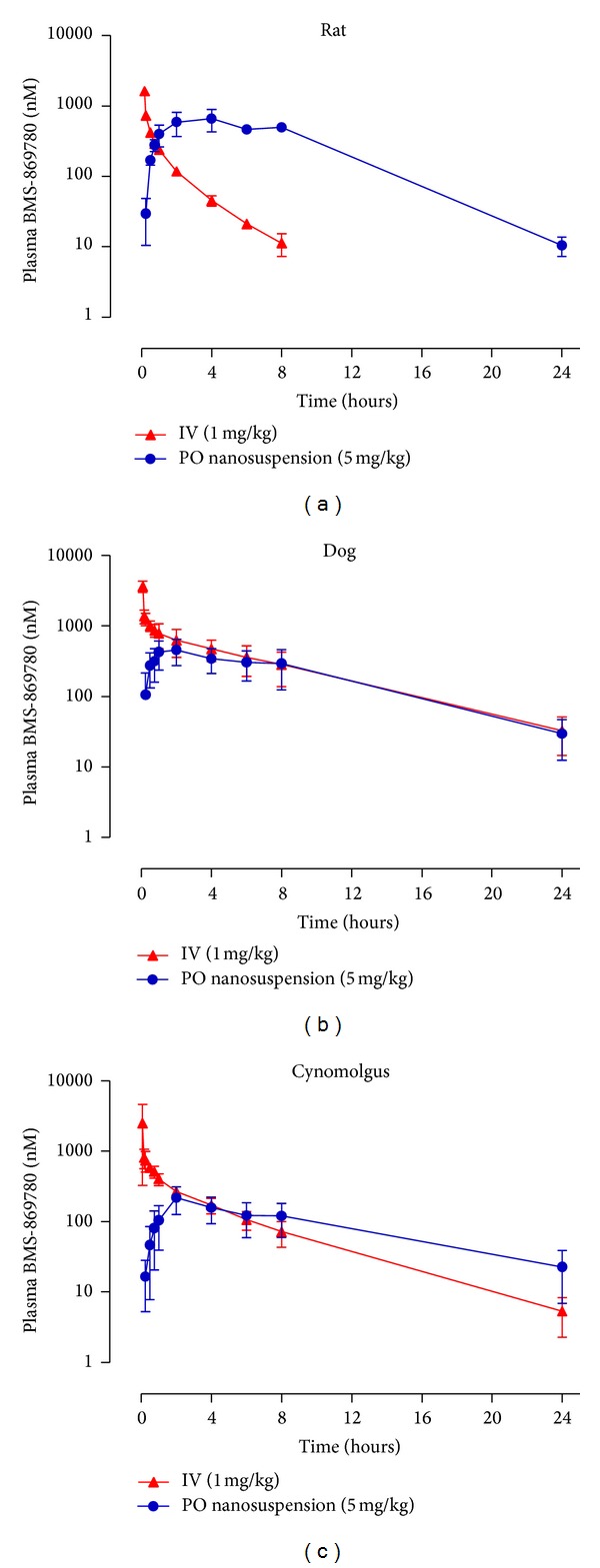
Pharmacokinetics (PK) of BMS-869780 across species for prediction of human PK. Rats, dogs, and cynomolgus monkeys were dosed with BMS-869780 intravenously (IV ▲) at 1 mg/kg or oral nanosuspension (PO ●) at 5 mg/kg. Plasma concentrations of BMS-869780 were determined for up to 24 hours after the dose. (a) Rat. (b) Dog. (c) Cynomolgus monkey. The derived PK parameters are summarized in Table 4.

To make a prediction of human PK for BMS-869780, the PK profiles for solution IV and nanoparticle suspension PO dosing of BMS-869780 were determined in rat, dog, and cynomolgus monkey (Figure 9; Table 4). Plasma exposure was readily detectable for 24 hours after dosing, and the average bioavailability of the nanoparticle suspension for the three species was 28%. Allometric scaling of the observed animal PK parameters was used to predict human PK parameters (Table 4) [70, 71]. From the predicted human PK, in combination with the PK/PD parameters from rat, a 10 mg/kg once daily dose (700 mg total) was predicted to achieve a steady-state AUC = 17.8 *μ*M·h, *C*_max⁡_ = 1.27 *μ*M, and corresponding brain A*β*1-42 ABEC = 25% (Table 3).

### 3.7. Off-Targets and Safety

**Figure 10:**
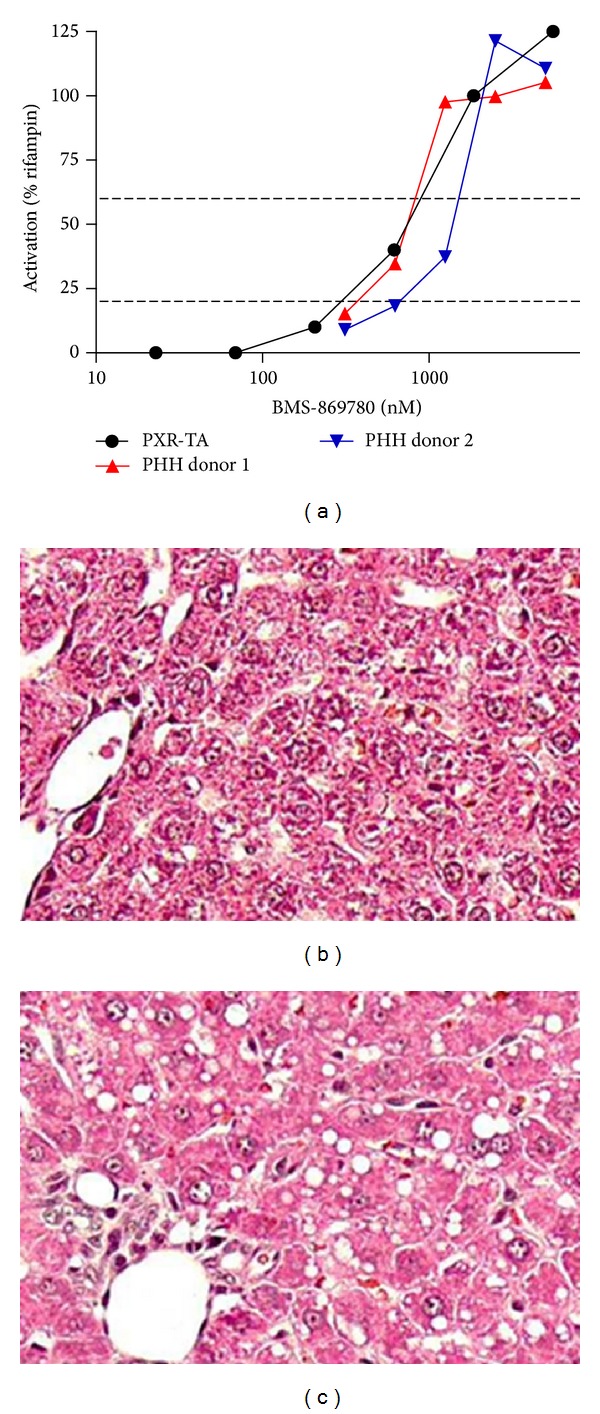
BMS-869780 caused PXR activation in vitro and lipidosis in rat liver. (a) PXR activation in the presence of BMS-869780 was evaluated in HepG2 cell cultures using a luciferase transcriptional reporter construct (●), or by assay of CYP3A4 mRNA levels in primary human hepatocyte (PHH) cultures from two individual donors (▲ and ▼). Activation in both assays is expressed as % relative to activation in the presence of rifampicin, 10 *μ*M, in parallel cultures. (b) Liver section from vehicle-dosed rats. (c) Liver section from rats given 4 daily doses of BMS-869780 at 100 mg/kg. A summary of the plasma BMS-869780 exposures from this experiment is shown in Table 5.

BMS-869780 was evaluated in a range of* in vitro* off-target activity assays. In one of these assays, pregnane-X-receptor transactivation (PXR-TA), BMS-869780 exhibited robust activity. In further experiments, BMS-869780 was shown to increase CYP3A4 mRNA expression in primary human hepatocytes. In both the PXR-TA and the primary human hepatocytes, transcription was activated at concentrations of 0.3 *μ*M and above (Figure 10(a)), suggesting potential activation of CYP3A4 metabolism and risk for drug-drug interactions in human at the exposures predicted to lower A*β*1-42.

As a preliminary evaluation of safety* in vivo*, male and female rats were given 10, 30, and 100 mg/kg BMS-869780 orally once daily for 4 days. Endpoints included brain A*β*1-42, as described above (Figures 7(d) and 7(e)), plasma PK on day 1 and day 3 (Table 4), and histopathology for duodenum, liver, and kidney. There was no loss of exposure between day 1 and day 3, consistent with a lack of autoinduction via PXR and/or lack of BMS-869780 metabolism by CYP3A in the rat. Duodenum and kidney histology were unchanged at all dose levels. However, liver exhibited macro- and microvesicular vacuolar degeneration consistent with a fatty acid change, likely lipidosis, at all doses (Figures 10(b) and 10(c)). In the 10 mg/kg dose groups, the lowest mean AUC = 17.5 *μ*M·h, and *C*_max⁡_ = 1.9 *μ*M, were commensurate with the target exposures determined for A*β*1-42 lowering (see Table 3). In contrast, even at the highest dose of 100 mg/kg, where exposures in excess of 200 *μ*M·h were achieved, there was no Notch-related effect on differentiation in the duodenum.

## 4. Discussion

The discovery and evaluation of BMS-869780 started with a high-throughput screen of the BMS compound inventory to identify selective inhibitors of A*β*1-42 in cell cultures and ended with predictions for human dose and exposure margins for off-target activity and safety. BMS-869780 is a GSM that decreased production of the longer peptides, A*β*1-40 and A*β*1-42, and increased production of the shorter peptides, A*β*1-37 and A*β*1-38. BMS-869780 did not significantly inhibit overall levels of A*β* production, APP-CTF processing, or Notch processing.

### 4.1. Potency and Mechanism of BMS-869780

BMS-869780 exhibited high potency (IC_50_ = 5.6 nM) for A*β*1-42 lowering in cell cultures, greater or equal in potency to the most potent GSMs reported [39, 43, 75, 76], and was robustly active* in vivo*, capable of lowering brain A*β*1-42 and A*β*1-40 in mice and rats by 75% or more. At the same time, levels of A*β*1-37 and A*β*1-38 were increased by BMS-869780, such that overall levels of A*β* remained essentially unchanged. The effect of BMS-869780 on *γ*-secretase therefore does not appear to involve inhibition. To evaluate the mechanism of BMS-869780 further, APP processing intermediates were evaluated both in H4-APPsw cell cultures and in rat brain. Levels of APP-CTF*β* and APP-CTF*α* were not affected by BMS-869780, in contrast to the GSIs BMS-698861 and BMS-299897, which caused robust accumulation. An unexpected observation emerged in the H4-APPsw cell line when it was found that GSI treatment, while causing robust APP-CTF*α* accumulation, did not result in APP-CTF*β* accumulation. This appears to be a quirk of the H4-APPsw cell line, possibly resulting from APP-CTF*β* degradation taking place predominantly through the proteasomal and lysosomal pathways, as recently reported [74]. In rat brain, *γ*-secretase inhibition caused robust increases in both APP-CTF*α* and APP-CTF*β*. In contrast, while BMS-869780 caused robust decreases in A*β*1-42 and A*β*1-40, there was no effect on APP-CTF*α* and APP-CTF*β* levels in rat brain. Thus, in the target organ, brain, BMS-869780 was demonstrated to act solely as a GSM, without inhibitory effects on *γ*-secretase.

The* in vivo* potency of BMS-869780 for brain A*β*1-42 lowering in rat and 3xTg mouse was evaluated in single dose time course experiments using a PK/PD indirect response model. This yielded* in vivo* IC_50_ values that were within a factor of 2-fold of the IC_50_ values for A*β*1-42 determined* in vitro*, when plasma protein binding was taken into account. In the 3xTg mouse, the* in vivo* and* in vitro* IC_50_ values were also within a factor of 2-fold when the effect of the presenilin-1 M146V allele on potency was additionally taken into account. The potency of BMS-869780 for A*β*1-42 lowering was ca. 3-fold less in cell cultures expressing the presenilin-1 M146V allele, consistent with previous reports that presenilin FAD mutants can affect the potency of GSMs [77, 78]. Thus, the* in vivo* activity of BMS-869780 in rodents corresponded well with its potency determined* in vitro*, suggesting that rodents would be predictive of activity in human.

### 4.2. Lack of Notch Inhibition by BMS-869780

The lack of effect of BMS-869780 on Notch processing was demonstrated using three approaches. First, a luciferase transcriptional reporter assay for Notch1 signaling was tested in cell cultures. For BMS-869780, the ratio of Notch to A*β*1-42 IC_50_s could not be precisely determined because of its weak activity in the Notch assays but was >1785-fold, based on an IC_50_ value >10 *μ*M for Notch. In the same assays, GSIs exhibited a wide range of Notch/A*β*1-42 IC_50_ ratios, with values of 13 and 723 for BMS-433796 and BMS-299897, respectively (Table 1). For GSIs, it has been shown that the absolute values of Notch/A*β*1-42 IC_50_ ratios for GSIs are strongly affected by APP substrate expression levels [61], and therefore cell culture data do not translate directly to Notch/A*β*1-42 margins* in vivo*. For example, the relatively Notch-selective GSI avagacestat exhibited a Notch/A*β*1-40 IC_50_ ratio of 193 [59], but the doses that achieved A*β* lowering without Notch-related side effects were more limited* in vivo* [19]. For BMS-869780, the lack of Notch-related side effects is expected based not only on the large* in vitro* Notch/A*β*1-42 IC_50_ ratio, but also on its noninhibitory mechanism of *γ*-secretase modulation. In rats, BMS-869780 did not cause any histological change in duodenum, specifically a lack of goblet cell metaplasia, even after four days of dosing at high exposures. The plasma exposure of BMS-869780 achieved in this experiment was more than 12-fold above the exposure required for lowering A*β*1-42 by 25% (A*β*1-42 ABEC = 25%). A wide variety of evidence from human genetics and transgenic APP mouse models suggests lowering of A*β*1-42 by 25% would be beneficial in AD [7]. Taken together, these data support the idea that GSMs such as BMS-869780 do not cause Notch-related side effects at doses predicted to cause sufficient A*β*1-42 lowering.

### 4.3. Human Predictions for BMS-869780

**Figure 11:**
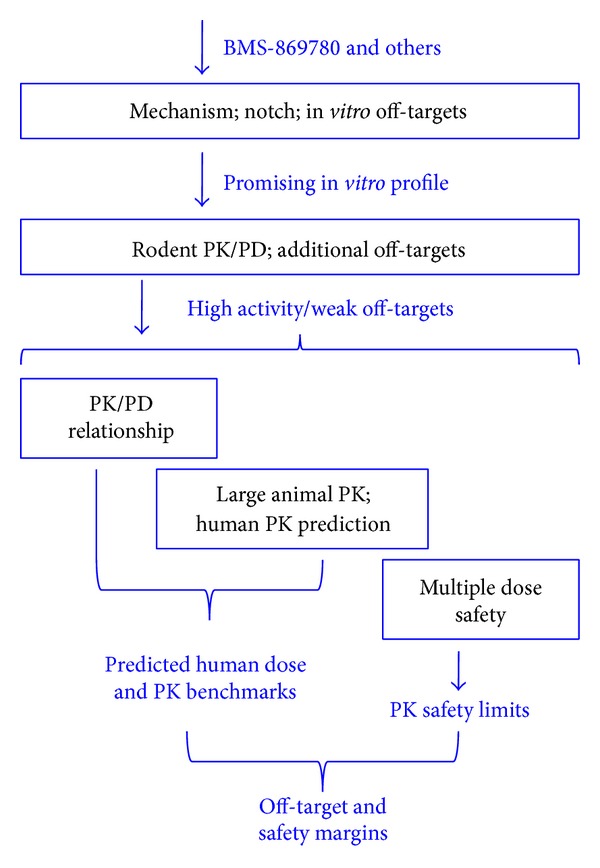
An overview of the characterization of BMS-869780 illustrates only the key steps in integration of data. Additional off-target and pharmaceutics evaluations necessary for decisions on individual compounds are not represented in this diagram.

To predict human dose, off-target, and safety margins, the PK/PD relationship determined in rodents was used as a guide, and a target A*β*1-42 ABEC = 25% was chosen, based on evidence from rodent models that 25% might ultimately translate to a significant effect in AD [7]. The assumption that human and rat PK/PD would be similar was supported by the concordance of the human* in vitro* IC_50_ with the* in vivo* IC_50_s determined in rat and mouse, as discussed above. The human PK parameters were then predicted through allometric scaling of PK in three species; rat, dog, and monkey, using the average bioavailability (*F* = 28%) achieved with a nanosuspension. The nanosuspension was chosen as a clinically relevant formulation with potential to enhance bioavailability. A dose of 700 mg was calculated to achieve brain A*β*1-42 ABEC = 25%, with associated AUC = 17.6 *μ*M·h and *C*_max⁡_ = 1.27 *μ*M. These AUC and *C*_max⁡_ values were then used as benchmarks to compare the A*β*1-42 lowering activity against* in vivo* side effects and* in vitro* off-target activities. BMS-869780 did not cause duodenal neoplasia, the characteristic Notch-related side effect observed in rats given GSIs, even after four days dosing that achieved AUC = 316 *μ*M·h and *C*_max⁡_ = 15.5 *μ*M (Table 5). This predicted a safety margin, specifically related to duodenal toxicity, including Notch-related side effects in rat, of at least 12-fold above the A*β*1-42 lowering exposure benchmarks. On the other hand, lipidosis in the liver was observed after four daily doses of 10 mg/kg with mean *C*_max⁡_ = 1.9 *μ*M and AUC = 17.5 *μ*M (Table 5), indicating no separation of hepatotoxicity from the A*β*1-42 lowering exposure benchmarks. Subsequent studies with other potent GSMs (not shown) did not exhibit hepatotoxicity under these conditions, suggesting an off-target mechanism of hepatotoxicity. BMS-869780 was evaluated in a wide range of* in vitro* off-target activity assays. In the case of the human PXR transcriptional reporter assay, BMS-869780 was found to be active at concentrations of 0.3 *μ*M and above. Further experiments confirmed the activation of CYP3A4 transcription in primary human hepatocyte cultures at similar concentrations, raising the possibility of metabolic induction and risk of drug-drug interactions at exposures required for A*β*1-42 lowering in human [79]. An overview of how experimental data were combined to determine off-target and safety margins is shown in Figure 11. In general, many GSMs exhibit poor drug-like properties, in particular high lipophilicity resulting in high active exposures and risk of systemic toxicity. Nevertheless, approaches for further optimization have been proposed, and the potential to improve drug-like properties has been demonstrated [80]. Alternatively, identification of new structural scaffolds might eventually lead to compounds with improved properties. Whether by optimization of current leads or new scaffolds, potentially the most useful guide for future compound design would be the availability of high resolution structures for GSM binding to *γ*-secretase.

In conclusion, BMS-869780 demonstrated the potential of the GSM approach, namely, the high potency, the robust translation of activity and mechanism* in vivo*, and the absence of a Notch-related side effect after multiple days of dosing at high sustained exposures. While liver toxicity and the high predicted dose of 700 mg caused studies of BMS-869780 to be discontinued, there was no evidence to suggest that liver toxicity or PXR activation were intrinsic to the GSM mechanism. In principle, therefore, an optimal combination of sufficient potency, PK, pharmaceutical properties, and off-target profile is within reach for testing a future GSM in Alzheimer's disease.

## Figures and Tables

**Table 1:** A*β* and Notch IC_50_ values.

Assay (cell line)	BMS-869780 (GSM)			BMS-299897 (GSI )			BMS-433796 (GSI)		
	IC_50_ (nM)	sd	*n*	IC_50_ (nM)	sd	*n*	IC_50_ (nM)	sd	*n*
A*β*1-42 (H4-APPsw)	5.1	2.2	13	0.47	0.61	322	0.16	0.08	256
A*β*1-40 (H4-APPsw)	24.1	7.7	7	1.3	1.2	93	0.19	0.14	20
A*β*1-42 (MEF PS1wt)	7.7	2.3	2						
A*β*1-40 (MEF PS1wt)	11	4	2						
A*β*1-42 (MEF M146V)	22	1.4	2						
A*β*1-40 (MEF M146V)	34	9	2						
mNotch1ΔE (luciferase)	>10000		3	340	108	147	2.1	1.0	256
mNotch1ΔE/A*β*1-42	>1785x			723x			13x		

**Table 2:** PK/PD model values from mouse and rat studies.

Parameter	Units	Mouse	CV (%)	Rat	CV (%)
PK parameters					
Kel	h^−1^	0.28	44.1	0.10	48.4
*V*/*F*	L/kg	1.94	48.3	1.94	43.8
Ka1 (10 mg/kg)	h^−1^			0.19	56.0
Ka1 (30 mg/kg)	h^−1^	0.11	29.8		
Ka2 (100 mg/kg)	h^−1^	0.046	30.0		

PD parameters					
*K*_OUT_	h^−1^	0.72	24.9	0.48	51.4
IC_50_	nM	3979	9.16	1892	26.4
*R*0	%	100	Fixed	100	Fixed
*I*_max⁡_		1	Fixed	1	Fixed
*K*_in_	%/h	72	Calculated	48	Calculated

Kel-first order rate constant for drug elimination; *V*-volume of distribution; Ka rate constant for drug absorption; *K*_OUT_-first order rate constant for degradation of A*β*1-42 or A*β*1-40; IC_50_-plasma concentration required for 50% inhibition of A*β*1-42 or A*β*1-40 production; *R* is the response in A*β*1-42 or A*β*1-40 levels, assumed 100% at time zero, *R*0; *I*_max⁡_-range of the response of A*β*1-42 or A*β*1-40 levels; *K*_in_-zero order constant for A*β*1-42 or A*β*1-40 production.

**Table 3:** Relationship between dose, plasma AUC, and A*β* ABEC.

Dose	Plasma AUC^0–24 hr^	Brain A*β* ABEC^24 hr^	
	(*μ*M*·*h)	A*β*1-42 (%)	A*β*1-40 (%)
Mouse 30 mg/kg PO solution (observed 0–24 hours)	45.9	30.9	n.d.
Mouse 100 mg/kg PO solution (observed 0–24 hours)	102	55.1	n.d.
Rat 10 mg/kg PO solution (observed 0–24 hours)	46.3	47.0	46.1
Rat 3 mg/kg PO solution (predicted steady state after 3 daily doses)∗	18.6	26.9	
Human 700 mg PO suspension (predicted steady state)∗	17.8	25	

*Dose predicted to achieve A*β*42 ABEC of ca. 25%.

**Table 4:** Summary pharmacokinetics observed in three species and predicted in human.

		Rat	Dog	Monkey	Human predicted
IV 1 mg/kg	Clearance (mL/min/kg)	24.3 ± 1.7	5.7 ± 1.7	15.2 ± 5.9	5.6
	AUClast (nM*·*h)	1484 ± 121	6640 ± 2257	2632 ± 926	
	Half-life (hour)	2.0 ± 0.3	5.2 ± 1.4	4.0 ± 0.5	13
	MRT (hour)	1 ± 0.2	6.6 ± 2.0	3.8 ± 0.7	
	Vss (L/kg)	1.8 ± 0.5	2.2 ± 0.8	3.7 ± 1.3	5.2

PO suspension 5 mg/kg	AUClast (nM*·*h)	3995 ± 964	4546 ± 2157	2031 ± 1038	
	*C*_max⁡_ (nM)	674 ± 227	487 ± 155	219 ± 94	
	*T*_max⁡_ (hour)	3.3 ± 1.2	1.7 ± 0.6	2.0 ± 0	
	*F* (%)	54%	13%	16%	28%

MRT-mean residence time; AUClast-area under the plasma concentration-time curve from zero time until the last quantifiable concentration.

**Table 5:** Summary of exposure in rat 4-day dosing experiment.

Dose (mg/kg)	Male		Female	
	Day 1	Day 3	Day 1	Day 3
	*C*_max⁡_ (*μ*M)			
10	2.0 (±0.4)	1.9 (±0.46)	2.9 (±0.4)	2.6 (±0.06)
30	6.1 (±1.8)	10.4 (±2.0)	8.2 (±1.7)	14.4 (±1.0)
100	7.7 (±0.9)	13.2 (±1.5)	10.6 (±1.0)	15.5 (±2.3)

	AUC (0–24 h) (*μ*M*·*h)			
10	20.2 (±4.8)	17.5 (±5.7)	41.0 (±5.6)	25.0 (±1.5)
30	95.2 (±19.9)	128.6 (±65.0)	152.2 (±21.0)	255.1 (±28.5)
100	167.5 (±14.5)	216.9 (±80.5)	205.9 (±43.2)	316.4 (±77.0)

	*T*_max⁡_ (h)			
10	5.0 (±2.0)	5.0 (±2.0)	7.0 (±2.0)	5.0 (±2.0)
30	8.0 (±0.0)	3.0 (±1.0)	5.0 (±2.0)	3.0 (±1.0)
100	19.0 (±9.0)	7.0 (±2.0)	24 (±0.0)	4.0 (±4.0)

Dose (mg/kg)	Terminal concentration day 4 (*μ*M)			
	Male		Female	

10	2.4 (±0.6)		3.0 (±0.3)	
30	8.9 (±1.9)		12.0 (±4.6)	
100	9.2 (±3.2)		13.7 (±0.9)	

## Abbreviations

ABEC: Area between baseline and A*β* effect-time curve
AD: Alzheimer's disease
APP: *β*-Amyloid precursor protein
APP-CTF: *β*-Amyloid precursor protein C-terminal fragment
AUC: Area under the plasma concentration-time curve
CYP3A4: Cytochrome P450 3A4 isozyme
DMEM: Dulbecco's modified Eagle's medium
ELISA: Enzyme-linked immunosorbent assay
FAD: Familial Alzheimer's disease
FRET: Förster resonance energy transfer
GAPDH: Glyceraldehyde-3-phosphate dehydrogenase
GSM: *γ*-Secretase modulator
GSI: *γ*-Secretase inhibitor
HTS: High-throughput screen
KO: Knockout (gene ablation)
dKO: Double knockout (gene ablation)
MEF: Mouse embryonic fibroblast
MRT: Mean residence time
NICD: Notch intracellular domain
PD: Pharmacodynamics
PK: Pharmacokinetics
PK/PD: Pharmacokinetic/pharmacodynamic relationship
PXR-TA: Pregnane-X-receptor transactivation assay
SEAP: Secreted alkaline phosphatase.
